# Maternal plasma fetuin-A levels in fetal growth restriction: A case-control study

**DOI:** 10.18502/ijrm.v17i7.4860

**Published:** 2019-07-31

**Authors:** Mujde Can Ibanoglu, Cem Yasar Sanhal, Seval Ozgu-Erdinc, Ozgur Kara, Aykan Yucel, Dilek Uygur

**Affiliations:** University of Health Sciences, Dr. Zekai Tahir Burak Women's Health Care, Education and Research Hospital, Ankara, Turkey.

**Keywords:** Fetal growth restriction, Fetuin-A, Pregnancy.

## Abstract

**Background:**

Higher Fetuin-A (FA) concentrations were found to be associated with obesity and there is an interest to the relation between maternal FA and pregnancy outcomes.

**Objective:**

In this study, our aim was to evaluate the association of maternal plasma levels of FA with fetal growth restriction (FGR).

**Materials and Methods:**

41 pregnant women with FGR and 40 controls were recruited in this case-control study between July and November 2015. At the diagnosis of FGR, venous blood samples (10 cc) were obtained for FA analysis.

**Results:**

Maternal plasma FA levels were significantly higher in fetal growth-restricted pregnant women compared with controls (19.3 ± 3.0 ng/ml vs 25.9 ± 6.8 ng/ml, p = 0.001). Area under receiver operating characteristic curve analysis of FA in FGR was 0.815 (95% confidence interval (CI): 0.718-0.912, p < 0.001). The maternal FA levels with values more than 22.5 ng/ml had a sensitivity of about 73.17% (95% CI: 56.79-85.25) and a specificity of about 82.5% (95% CI: 66.64-92.11) with positive and negative predictive values of about 81.08% (95% CI: 64.29-91.45) and 75% (95% CI: 59.35-86.30), respectively. Therefore, the diagnostic accuracy was obtained about 77.78%.

**Conclusion:**

The results of this study show higher maternal plasma levels of FA in FGR. Further studies are needed in order to demonstrate the long-term effects of FA in pregnancies complicated with FGR and early prediction of FGR.

## 1. Introduction

Fetal growth restriction (FGR) is reported in 7-15% of all pregnancies (1), the condition is suggested as one of the most common problems in perinatal medicine. There are many debates about FGR, where a universal consensus definition has yet to be gained. American College of Obstetricians and Gynecologists describes FGR as “fetuses with an estimated fetal weight less than the 10th percentile for gestational age” (2). Recently, a consensus definition of intrauterine growth restriction was published defining early and late FGR together with cut-off values for the involved parameters (3). Postnatal abnormal neurological development, and neonatal morbidities such as polycythemia, intraventricular bleeding, and other metabolic disorders impose further importance on FGR (4-6).

There has also been an interest in the relation between Fetuin-A (FA) (Human fetuin-A/alpha2-Heremans-Schmid glycoprotein) and FGR pregnancies. Initially, FA levels were reported to increase with gestational age (7). Higher FA concentrations were found to be associated with obesity and atherogenesis (8). Moreover, gestational diabetic women exhibited elevated levels of FA compared to pregnant women with normal glucose metabolism (7). However, FA has been known to regulate the function of insulin in adipocytes and skeletal myocytes (9) and reported to involve in the inhibition of calcification by impeding the calcification-inducing effects of major proteins that promote cardiac valvular calcifications (10). The origin of its serum isoform is the hepatocytes, and its hepatic phosphorylation decreases the transduction of the signal of the insulin receptor that may improve insulin resistance during pregnancy (9, 11). FA was also defined as a negative inflammatory mediator (11). In addition, it was shown that FA significantly increased in pregnant women with preeclampsia (12-14). Moreover, FA may predict the success of in-vitro fertilization procedures (14). It has been suggested that besides FA, insulin and insulin-like growth factors have a role in the growth of the fetus (15).

In this study, we aimed to evaluate the role of maternal plasma levels of FA in pregnancies complicated with FGR.

## 2. Materials and Methods

Between July and November 2015, all pregnant women who were referred to the Perinatology Department of tertiary referral hospital and had the diagnosis of FGR were enrolled in the trial. The definition of FGR was based on the estimated fetal weight below the 10th percentile on ultrasound, together with birth weight below the 10th percentile of the standard growth curve (2, 16). Age, body mass index, gravidity, and gestational age-matched pregnant women who were routinely followed up were included into the control group. Any fetus with chromosomal abnormalities, multiple pregnancies, singletons with systemic disease including hypertension (systolic and diastolic arterial tension > 140/90 mmHg, respectively), diabetes mellitus (according to positive 50 gr and 100 gr oral glucose tolerance test), alcohol users or smokers, any chronic drug users were excluded from the study. At the diagnosis of FGR, venous blood samples (10 cc) were obtained in sterile conditions into ethylenediaminetetraacetic acid red tubes. The samples were centrifuged at 4000 rpm for 10 min. The samples were stored at -80°C until the time of analysis. Calculation of plasma FA levels was performed by a sandwich enzyme-based technique (BioVendor Laboratory Medicine Inc., Brno, Czech Republic) (17). Plasma FA concentration values were expressed as ng/ml.

### Ethical consideration

This study was conducted according to the Declaration of Helsinki (18). The institutional review board of the University of Health Sciences, Zekai Tahir Burak Women's Health Care, Education, and Research Hospital approved the protocol of this case-control study (28/10/2014 #26) and all participants gave a written informed consent.

### Statistical analysis

IBM SPSS (Statistical Package for the Social Sciences) Version 23 (IBM Corp., Armonk, NY, USA) was used for the statistical analysis. The Kolmogorov-Smirnov test was used to evaluate the normality of the variables of the groups. Parametric data were evaluated using the independent two-sample t-test, and non-parametric data were compared using the Mann-Whitney U test. The screening efficiency of FA was evaluated by the receiver operating characteristic (ROC) analysis. The cut-off value obtained by ROC curve analysis was employed in the measurement of sensitivity, specificity, positive and negative predictive values, and diagnostic accuracy. A two-tailed p-value < 0.05 was considered statistically significant.

## 3. Results

The demographic features of the participants are depicted in Table I. There were 41 pregnant women with FGR and 40 controls. No difference was found between the study group and controls in terms of age, body mass index, gravidity, parity, and gestational week. The FGR was characterized by lower neonatal birth weight and Apgar score at min 5 (p < 0.001).

**Figure 1:**
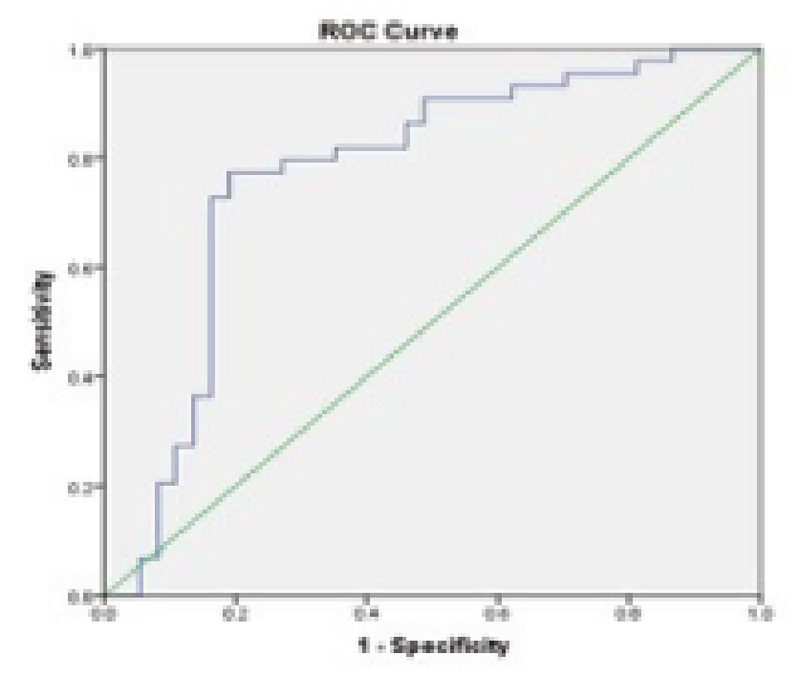
The area under the receiver operating characteristic curve for Fetuin-A in fetal growth restriction.

Maternal plasma FA levels significantly increased in FGR group compared to controls (19.3 ± 3.0 ng/ml vs 25.9 ± 6.8 ng/ml, p = 0.001) (Table I). Figure 1 shows the area under ROC curve (AUC) analysis of FA in FGR which is 0.815, (95% confidence interval (CI): 0.718-0.912, p < 0.001). The maternal FA levels with values more than 22.5 ng/ml had a sensitivity of about 73.17% (95% CI: 56.79-85.25) and a specificity of about 82.5% (95% CI: 66.64-92.11) with positive and negative predictive values (PPV and NPV) of about 81.08% (95% CI: 64.29-91.45) and 75% (95% CI: 59.35-86.30), respectively. Therefore, the diagnostic accuracy was about 77.78%.

**Table 1:** Demographic features and the results of patients


	**FGR (n = 41)**	**Control (n = 40)**	P-value*
Age (yr)	25.9 ± 6.0	27.2 ± 5.2	0.101
BMI (kg/m${}^{2}$)	28.2 ± 4.2	28.0 ± 3.2	0.765
Gravida (number)	1.9 ± 1.2	2.0 ± 1.2	0.535
Parity (number)	0.7 ± 0.9	0.7 ± 0.7	0.880
Gestational age at blood sampling (wk)	37.0 ± 2.9	36.8 ± 3.4	0.875
Birthweight (gr)	2401 ± 413	3200 ± 546	< 0.001
Apgar score at min 5	9.7 ± 0.6	8.7 ± 0.8	< 0.001
Fetuin-A level (ng/ml)	25.9 ± 6.8	19.3 ± 3.0	0.001
Umbilical Artery S/D ratio	2.4 ± 0.64	2.12 ± 0.29	0.001
Data presented as mean ± SD; Mann Whitney U test			
FGR: Fetal growth restriction			
BMI: Body mass index			
P < 0.05 indicates significant difference			

## 4. Discussion

There was no statistically significant difference between the FGR group and the control group in terms of age, body mass index, gravidity, parity, and gestational age at blood sampling. However, Fetuin-A level was found higher in the FGR group compared to the control group. Maternal factors as hypertensive disorders, renal disease, smoking, alcohol use, severe nutritional deficiencies; fetal factors as multiple gestations, infections, aneuploidy or structural abnormalities, and finally placental abnormalities have been traditionally defined as the causes of FGR (19). It is relatively straight forward to enlighten the underlying reason of an FGR case with placental insufficiency (by using Doppler ultrasound), aneuploidy (by karyotyping), infections (by Polymerase chain reaction analysis), and structural abnormalities (by ultrasound) (2, 20). However, in some instances, efforts regarding the etiology of FGR do not reveal significant results. There is a tendency to use the term “constitutionally small or small for gestational age (SGA)” for the cases that do not present with advanced forms of smallness (estimated fetal weight < 3${}^{rd}$ centile, or signs of fetoplacental Doppler adaptation, defined as abnormal uterine artery (UtA) Doppler, and “FGR” for the cases with the mentioned features (21). However, the pathophysiological basis of these clinical forms has not been truly understood, and their associations with long-term outcome have not been completely defined yet. In addition, recent data reporting lower two-year neurodevelopmental scores for full-term SGA infants (without placental insufficiency) compared to the normal-sized babies challenged the concept that SGA fetuses with normal umbilical artery Doppler are “constitutionally small” but otherwise completely normal (22). In the present study, we used the definition for FGR that was suggested by the American College of Obstetricians and Gynecologists, in which the diagnosis is mainly performed by the estimated fetal weight and/or birthweight percentiles. The relationship between FA and FGR was investigated by a limited number of trials. Briana and colleagues examined the circulating levels of human FA in pregnant women and fetuses from FGR and appropriate-for-gestational-age pregnancies. They found that plasma FA values showed no difference between FGR cases and appropriate-for-gestational-age controls (23). The authors also mentioned that maternal and fetal FA concentrations did not differ from each other and they had a positive correlation that pointed at the likelihood of passive transplacental transfer (23). Conversely, Karamessinis and co-worker found the post-translational modifications of the FA in umbilical cord plasma of FGR neonates by showing the single- and heavy-chain forms of FA that did not contain the normally present O-linked sialic acids. They concluded that prominent defects in glycosylation/sialylation of FA which were shown in their study might be the reason for the functional impairment of FA, leading to deficient fetal growth (24).

In another well-designed study, Gomez and colleagues declared that extravillous cell viability and invasion was reduced by FA. Moreover, elevated plasma FA values were more frequently observed in preeclampsia groups than control cases, even after we controlled for diabetes mellitus, hypertension, and obesity (25).

The findings of the present study, indicating elevated plasma FA levels in pregnancies complicated by FGR, also supported the view that high concentrations of FA ended in a reversal of the invasiveness of extravillous trophoblast cells that was enhanced by growth factors. Today, the association between FA and metabolic syndrome (understood as a disease entity) is strongly proved (26). In addition, the rise in plasma values of FA up to 10 times following hypoxic changes have been reported in some animal trials, which may be contributed by injured-cell-derived high-mobility group box-1 protein in the expression of this substance (27, 28). It was also announced that plasma FA levels were increased in study participants with brain ischemic injury. The plasma FA levels were supported positively with high levels of low-density lipoprotein cholesterol (29, 30). In our point of view, these common findings suggest further investigations for the possible mutual mechanisms - in addition to the inhibition of insulin receptor tyrosine kinase which is the main function of FA - for FGR and trauma, a stroke which may give extra hint to the pathogenesis and management of FGR.

Our study has some limitations which suggest that a more comprehensive study is needed to draw a more concrete conclusion in terms of FA levels in the normal population and pregnant women with FGR, and to create percentile ranks for gestational weeks as well. As being a negative acute phase reactant, research of FA in relation with other frequently used markers for inflammation may reveal the inflammation status of the disorder (31). And finally, the detection of the umbilical cord or neonatal plasma FA levels would denote additional data about the issue.

## 5. Conclusion

The results of this study show higher maternal plasma levels of FA in FGR. Further studies are needed in order to demonstrate the long-term effects of FA in pregnancies complicated with FGR and early prediction of FGR.

##  Conflict of Interest

The authors report no conflicts of interest.
